# A molten salt-based nitridation approach for synthesizing nanostructured InN electrode materials†

**DOI:** 10.1039/d0ra07172b

**Published:** 2020-10-12

**Authors:** Gani Purwiandono, Kazuhiro Manseki, Takashi Sugiura

**Affiliations:** The Graduate School of Natural Science and Technology, Gifu University 501-1193 Japan kmanseki@gifu-u.ac.jp; Department of Chemistry, Universitas Islam Indonesia 55584 Indonesia

## Abstract

Single-phase InN nanocrystals were synthesized for the first time by a molten salt-based nitridation approach using InCl_3_ and LiNH_2_ as indium and nitrogen sources, respectively. A molten salt, KCl–LiCl, during nitridation, enabled us to obtain InN nanocrystals at relatively low temperatures ranging from 400 °C to 500 °C. SEM and HR-TEM measurements coupled with XRD data revealed that InN nanocrystals were formed with average grain sizes of approximately 50–60 nm. Notably, the photoelectrochemical cell fabricated using the InN nanocrystals synthesized at 450 °C exhibited a photocurrent response under light irradiation from 400 nm to 880 nm. The precise control of the growth of InN particles using our synthetic approach provides opportunities for developing versatile nitride nanocrystals.

## Abstract 2

Single-phase InN nanocrystals were synthesized for the first time by a molten salt-based nitridation approach using InCl_3_ and LiNH_2_ as indium and nitrogen sources, respectively.

## Graphical abstract

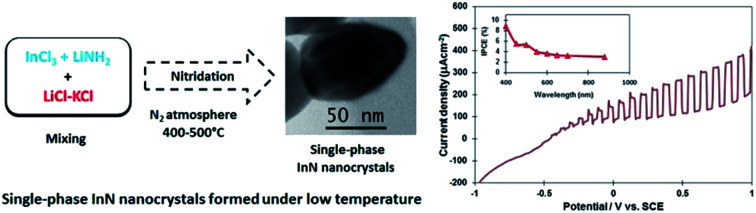

## Introduction

Indium nitride (InN) has been regarded as an attractive semiconductor for device applications such as light-emitting diodes (LEDs) and infrared detectors.^1–5^ Moreover, the narrow band gap, typically from 0.6 eV to 0.9 eV, enables the detection of its spectral range in the near-infrared region.^6–13^ In growing pure InN, the main difficulty lies in its thermal decomposition, which becomes significant above 600 °C. In addition, high-temperature treatment causes the inclusion of impurities, especially oxygen atoms.^14,15^

Much attention has been given to the synthesis of single-phase InN using several methods, such as molecular beam epitaxy (MBE), metal organic chemical vapor deposition (MOCVD), and plasma and sputtering techniques.^16–23^ However, few reports are available on nitridation reactions for growing nanoscale InN crystals.

Jung *et al.*^24^ reported the gas-phase nitridation synthesis of InN with NH_3_ gas at 600 °C. InN particles with a diameter of approximately 300 nm were obtained. Our group has previously demonstrated the formation of InN at 400 °C using In_2_O_3_ as an indium source in indium flux under N_2_ gas flow. Particulate InN crystals with a diameter of approximately 800 nm were obtained.^25^ These studies have demonstrated the effect of raw materials (In_2_O_3_ and LiNH_2_) on the morphology of InN crystals. However, none of the above-mentioned studies have investigated the morphology control of InN particles at the nanoscale and their photoelectrochemical properties.

We have successfully synthesized 2D hexagonal-shaped GaN nanoplates through the reaction of GaCl_3_ and LiNH_2_ using LiCl as the molten salt.^26^ The use of LiCl as the molten salt was found to accelerate the homogenous GaN formation at the nanoscale. Photoelectrochemical analysis of the GaN electrode revealed that the hexagonal-shaped GaN electrode exhibited an anodic photocurrent that was higher by a factor of 2 compared to that of the random-shaped GaN. With regards to the InN photo-electrode, Lindgren *et al.*^27^ reported an incident photon-to-electron conversion efficiency (IPCE) of InN film of up to 2% at 350 nm, whereas no photocurrent response was observed in the visible and near-IR regions.

The particular importance of our molten salt-based synthesis lies in the size control of InN particles at a nanoscale, which is a new concept for synthesizing InN nanocrystals. Our previous study on LiCl-based GaN synthesis showed that the Cl^−^ anions of LiCl can suppress the crystal growth of GaN through the Cl^−^ coordination to Ga(iii).^26^ Based on the results, we anticipated that InN nanocrystals could be synthesized using LiCl-containing molten salts. In this paper, we use a KCl–LiCl mixture with eutectic composition of 60% mol LiCl and 40% mol KCl that has a melting point of 353 °C (as shown in Table S1†^28^), taking into consideration that the molten salts for nitridation reactions of InN should have a melting point that is lower than the synthesis temperature at 400–500 °C.^25,29^

Herein, we present, for the first time, the effect of molten salt on the nitridation synthesis of single-phase InN nanocrystals. The InN nanocrystals were successfully obtained at relatively low temperatures ranging from 400 °C to 500 °C. In addition, the photoelectrochemical cell fabricated using the InN electrodes exhibited a photocurrent response under visible and near-IR light irradiation. Importantly, our methodology for controlling the growth of InN nanocrystals using molten salt-assisted nitridation provides a new approach to create various nitride nanocrystals and their electrodes for photo-energy conversion.

## Experimental section

### Synthesis of InN nanocrystals

InCl_3_ (0.664 g, 1 mol), LiNH_2_ (0.482 g, 7 mol), LiCl (0.153 g, 0.8 mol), and KCl (0.179 g, 1.2 mol) were mixed in a graphite crucible (inner diameter: 55 mm, length: 309 mm, SUS 316) under N_2_ atmosphere (50 mL min^−1^). The mixture was preheated at 150 °C for 30 min. The nitridation reactions were then carried out at 350 °C, 400 °C, 450 °C, 500 °C, and 550 °C for 2 h under N_2_ atmosphere. The ramping rate for the nitridation reaction was set to 10 °C min^−1^, and the cooling rate was set to 2 °C min^−1^. After cooling, the products were washed with 1 M HCl and ethanol to obtain InN powder samples.

### Preparation of InN photoelectrodes

InN/titanium paste electrodes were prepared to examine the photoelectrochemical properties of InN films with a thickness of 0.5 μm. To prepare InN electrodes, the as-synthesized InN powder was mixed with a 2-butanol solution (20 wt%, Wako, 99%) for 24 h. The paste was deposited on a Ti substrate (Nilaco) by the doctor blade technique using scotch tape as a spacer. Prior to deposition, the Ti substrate was cleaned with an etching solution (a mixture of 7 mL distilled water, 5 mL HNO_3_ (Nacalai Tesque, 60%), and 1 mL HF (Wako, 46%)). The heat treatment was performed at 350 °C for 2 h under a N_2_ atmosphere. Then, 5 mL of a NaOH (5 mM, Kanto, 97%) solution was added to 20 mL of a Co(NO_3_)_2_·6H_2_O (5 mM, Wako, 99.5%) solution, and a portion of this mixture was dropped onto the InN film. Subsequently, heat treatment was performed at 350 °C for 2 h under a N_2_ atmosphere.

### Structural characterization of InN powder

The structure of the InN powder was analyzed by X-ray diffraction (XRD, Rigaku Ultima II/OC) with CuK_α_ radiation and transmission electron microscopy (TEM, JEOL, JEM-2100). The morphology of the InN particles was observed by scanning electron microscopy (SEM, Hitachi, S-4800). The elemental analysis of the samples was performed *via* X-ray photoelectron spectroscopy (XPS, ULVAC, Quantera SXM). The specific surface areas of the InN powders were determined using a gas sorption analyzer (Micromeritics Tristar II 3020). The absorbance measurements were carried out on a UV-vis spectrophotometer (Hitachi, U-4000).

The Debye–Scherrer equation was applied to determine the crystallite size of InN powder using eqn (1). *D*, *λ*, *β* and *θ* correspond to the crystallite size, wavelength of X-ray radiation, full width at half maximum (FWHM), and diffraction angle, respectively.1
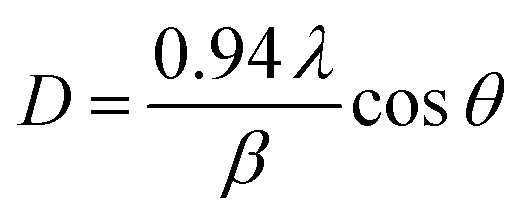


### Photoelectrochemical measurements

Photoelectrochemical measurements were performed with a three-electrode cell consisting of the InN photoanode, saturated calomel reference electrode, and platinum counter electrode using 1 M Na_2_SO_4_ (50 mL). H_2_O_2_ (1 mL, 30% concentration) was also added to the electrolyte.^28,30^ The current–voltage characteristics were obtained using a potentiostat under intermittent UV light irradiation with a light intensity of approximately 120 mW cm^−2^ (Xe lamp with a 64R filter). Mott–Schottky analysis was applied to determine the donor density (*N*_D_) of the InN electrodes using eqn (2). *E*, *E*_FB_, and *c* correspond to the applied potential, flat band potential, and space charge capacitance in the electrode, respectively. *T*, *k*, *e*, *ε*_o_, and *ε* are the absolute temperature, Boltzmann constant, elemental charge, vacuum permittivity, and relative permittivity, respectively. A relative permittivity of 15.3 F m^−1^ was used to obtain the *N*_D_ value using eqn (3). The Mott–Schottky plots were obtained using a VersaSTAT3 potentiostat. The measurements were performed using a 1 M Na_2_SO_4_ solution at a given bias potential under dark conditions. The measured frequency was 1 kHz.2
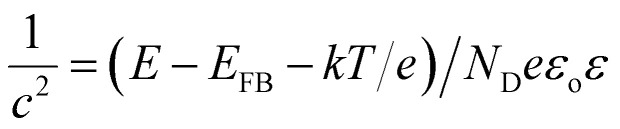
3
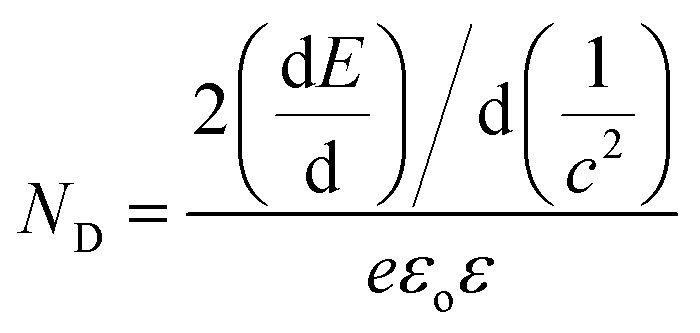


The IPCE measurements were performed using a three-electrode configuration with an InN working electrode, a Ag/AgCl reference electrode, and a platinum counter electrode. The electrodes were immersed in the same electrolyte as mentioned above. The quantum efficiency was recorded at an applied potential of 1.0 V *vs.* Ag/AgCl. A metal halide lamp was used with bandpass cut filters (400, 450, 500, 550, 600, 650, 700, and 880 nm). The incident light intensities were 3.0–8.0 mW cm^−2^ for each filter to obtain the IPCE using eqn (4).4
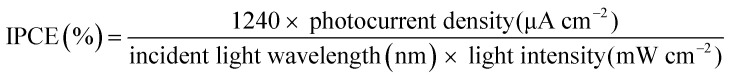


## Results and discussion

### Synthesis of InN nanocrystals

**Fig. 1:**
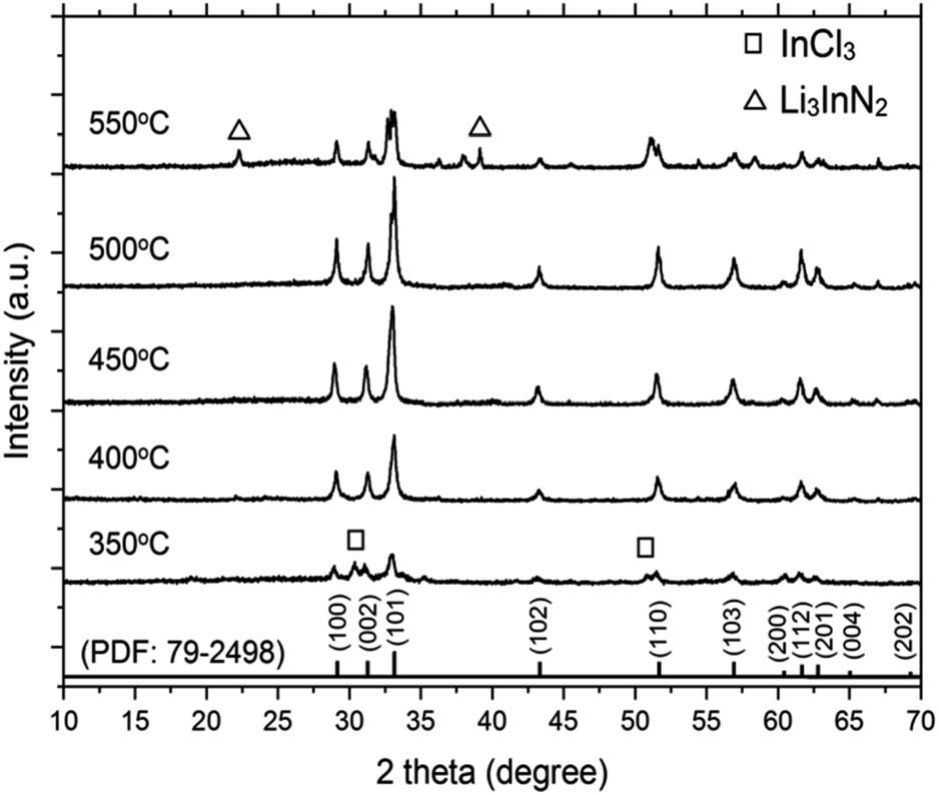
XRD patterns of InN synthesized using InCl_3_, LiNH_2_, and LiCl–KCl. The reactions were carried out at 350 °C, 400 °C, 450 °C, 500 °C, and 550 °C.

Fig. 1 shows the XRD pattern of the synthesized InN powders using LiCl–KCl as the molten salt at various temperatures. The diffraction peaks of the InN products can be indexed to the JCPDS data (PDF: 79-2498). At temperatures of 400 °C, 450 °C, and 500 °C, no peaks of byproducts were detected, indicating the formation of single-phase InN crystals for all samples. A control reaction at 350 °C, which is lower than the melting point of LiCl–KCl (353 °C), was also carried out. Peaks of InN and InCl_3_ were observed as byproducts, suggesting that LiCl–KCl could act as a reactant at temperatures below the melting point. Furthermore, at higher reaction temperatures (400 °C and 450 °C), the intensity of all the InN diffraction peaks increased when compared to that at 350 °C. These observations indicate that LiCl–KCl plays a crucial role in facilitating the single-phase crystal growth of InN. However, this trend was only observed up to 500 °C. Above 550 °C, the peak corresponding to the (101) plane significantly decreased, and Li_3_InN_2_ was concomitantly formed. This result was probably due to the decomposition of InN. The crystallite sizes along the (101) plane of InN are presented in Table 1. The largest crystallite size of 53 nm was obtained from the sample prepared at 450 °C.

**Table 1:** Reaction yields and structure characterization of InN nanocrystals

InN sample	Reaction yields (%)	Specific surface area (m^2^ g^−1^)	Average grain size (nm)	Crystallite size^a^ (nm)
400 °C	42	13	49	41
450 °C	72	12	59	53
500 °C	62	14	51	50

a Determined using the XRD data of InN corresponding to the (101) plane.

To characterize the product, XPS spectra were obtained (Fig. S1†). For the reaction temperatures of 400 °C, 450 °C, and 500 °C, the binding energies of In 3d_5/2_ and N 1s were 443.6 and 396.5 eV, respectively. The binding energies of In 3d_5/2_ and N 1s were consistent with the reported values for InN, and no peaks corresponding to Cl 2p and Li 1s originating from byproducts were observed.^29,31^ For samples prepared at 350 °C and 550 °C, peaks at 57.5 eV and 198.9 eV corresponding to Li 1s and Cl 2p, respectively, were detected.

**Fig. 2:**
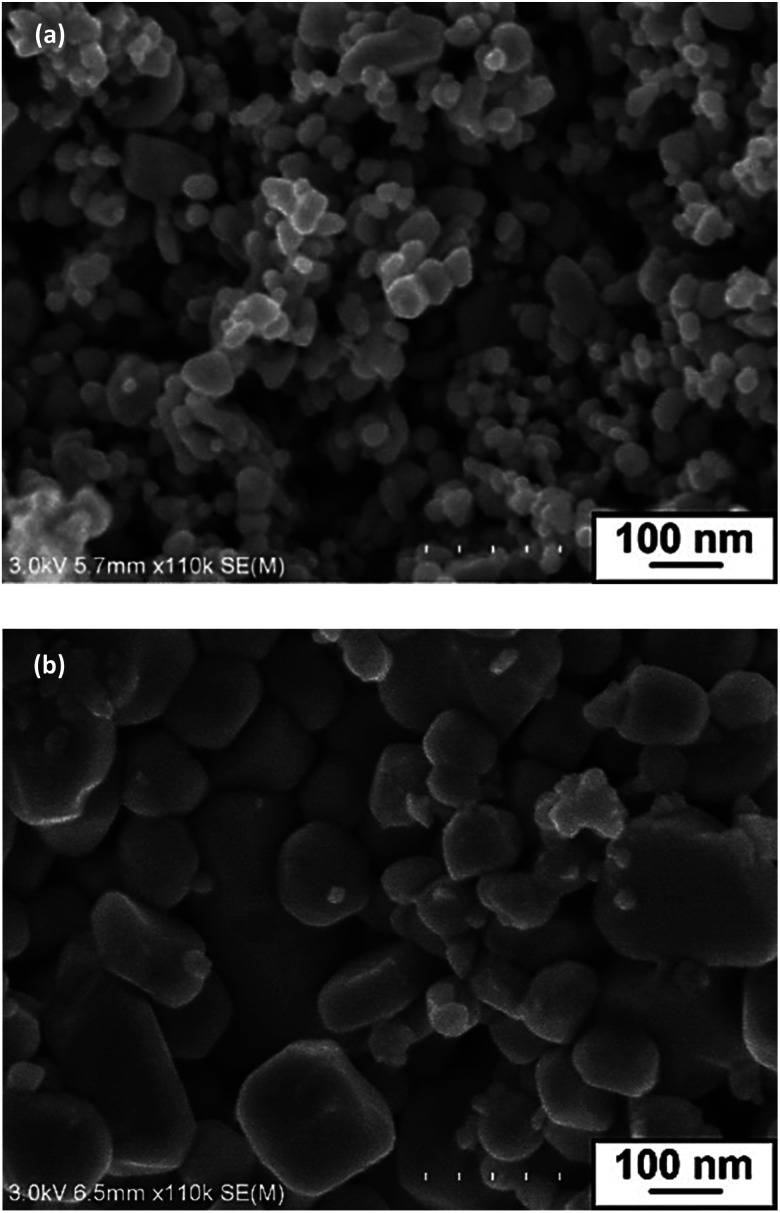
SEM images of InN samples prepared at 450 °C by the reaction of (a) InCl_3_, LiNH_2_ and LiCl–KCl and (b) InCl_3_ and LiNH_2_.

SEM images of InN powder samples formed at 350, 400, 450, 500, and 550 °C are shown in Fig. 2a and S2.† The SEM images clearly indicated that with the use of LiCl–KCl as the molten salt, InN nanocrystals with grain sizes smaller than 100 nm were formed at 400 °C, 450 °C, and 500 °C (Table 1). The largest grain size was 59 nm, which was measured for the sampled prepared at 450 °C. The BET specific surface areas of these three samples had comparable values of approximately 12–14 m^2^ g^−1^, as summarized in Fig. S3.†

The reaction yields and crystallite sizes for the (101) plane of InN synthesized at different temperatures are also shown in Table 1. The crystallite sizes along the (101) plane of InN synthesized at 400, 450, and 500 °C were 41 nm, 53 nm, and 50 nm, respectively. The reaction yields increased with increasing reaction temperature from 400 °C to 450 °C. The highest reaction yield (72%) for the (101) plane was calculated for InN synthesized at 450 °C.

To understand the role of molten salt in crystal growth, we synthesized InN without LiCl–KCl (the XRD pattern and SEM images are presented in Fig. S4 and S5†). In contrast to that of the molten salt synthesis, the XRD pattern exhibited peaks that were assigned to the byproduct InLi (Fig. S4†). The SEM images indicated that the average grain size of InN synthesized without molten salt was significantly larger than that obtained using the molten salt (Fig. S5†); Fig. 2b shows one of such SEM images (450 °C sample). Considering that a random-shaped morphology with an average grain size of 200 nm was obtained, it is likely that the LiCl–KCl molten salt contributed to the suppression of InN growth, leading to the size reduction in the InN formation.

**Fig. 3:**
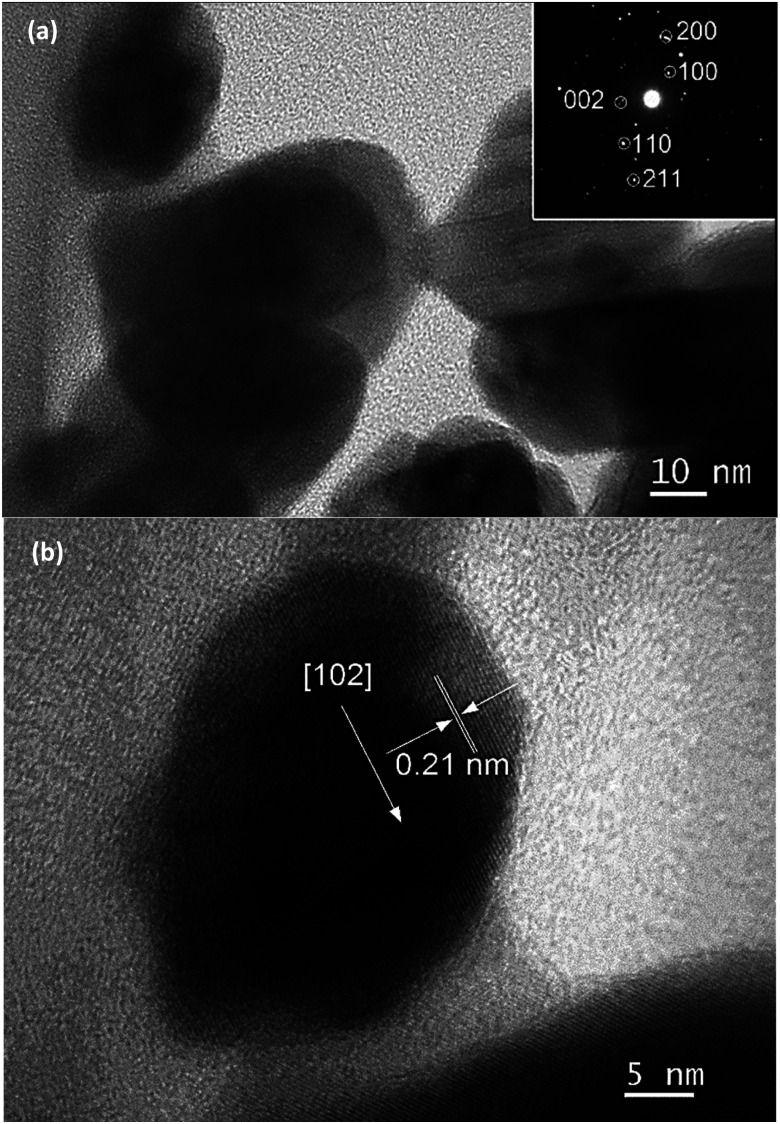
TEM images of (a) InN nanocrystals synthesized at 450 °C for 2 h and (b) lattice fringe of (100) plane.

The high resolution transmission electron microscope (HR-TEM) images of InN nanocrystals synthesized at 450 °C are also presented in Fig. 3a. A lattice fringe of 0.30 nm corresponding to the spacing of the (100) plane was observed (Fig. 3b). Analysis of the selected area electron diffraction (SAD) pattern indicated that single-phase InN nanocrystals were present.

The plausible reactions for the formation of InN nanocrystals are shown below.

5 2InCl_3_ + 6LiNH_2_ → 2Li_3_InN_2_ + 3Cl_2_ + 6H_2_ + N_2_

6 2Li_3_InN_2_ + 3Cl_2_ → 2InN + 6LiCl + N_2_

In the first step (eqn (5)), the mixture of reactants (InCl_3_ and LiNH_2_) and the LiCl–KCl salt were uniformly heated, resulting in the decomposition of LiNH_2_ at 370 °C to form Li_3_InN_2_ particles. It is presumed that the InN nanocrystals were formed by the decomposition of Li_3_InN_2_ in the second step (eqn (6)). Single-phase InN without any byproduct was observed at temperatures from 400 °C to 500 °C. Upon increasing the reaction temperature to 550 °C, Li_3_InN_2_ was produced as a byproduct, as shown in the XRD pattern. The decreased intensity of InN indicated that InN started to decompose at approximately 550 °C.

**Fig. 4:**
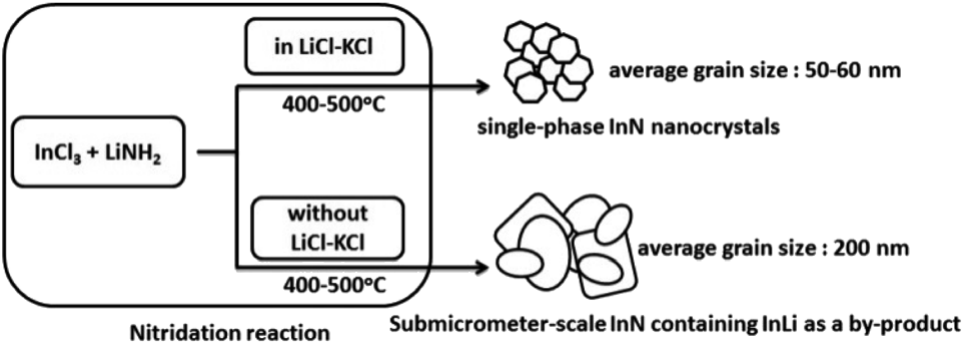
Schematic diagram of the effect of molten salt on the crystal growth of InN.

In a previous study, we successfully synthesized GaN nanoplates using LiCl as the molten salt. The molten salt could accelerate the homogenous formation at the nanoscale during the nucleation and crystal growth of GaN particles.^26^ The effect of LiCl–KCl on the crystal growth of InN can be explained as follows (Fig. 4): excess Cl^−^ anions in the nitridation process (eqn (6)) can interact with the (100), (002), and (101) surfaces. This phenomenon probably leads to the suppression of crystal growth, producing InN nanocrystals with a narrow size distribution. However, without the use of LiCl–KCl as the molten salt, it is likely that the lack of Cl^−^ anions resulted in the formation of random-shaped particles.

A few solid-state nitridation reactions have been reported to promote the formation of InN. One reported reaction condition includes the use of NaNH_2_ as a solid-nitrogen source for LiInO_2_; InN aggregates with a size range of 50–300 nm were synthesized at 240 °C.^30,32^ Most importantly, our nitridation using a molten-salt is unique in that the reaction allowed us to form InN with a narrow size distribution, as shown in Fig. 2a.

In order to determine the band gap, the absorption spectra of InN thin films were also measured from 300 to 2500 nm, as shown in Fig. S6.† The band gaps of InN synthesized at 400, 450, and 500 °C were estimated to be 0.8–0.9 eV, which is consistent with the reported value for InN.^11–13^

As mentioned above, we have previously synthesized GaN nanoplates through the reaction of GaCl_3_ and LiNH_2_ using LiCl under N_2_ atmosphere.^26^ In addition, Carter *et al.*^33^ have reported the nitridation reaction of aluminum nitride (AlN) from AlCl_3_ in trimethylphenylammonium chloride (TMPAC) as a molten salt under NH_3_ gas. The polycrystalline AlN was successfully synthesized at 900 °C. Our synthetic protocol using a molten salt approach could have significant potential for synthesizing various metal nitride nanocrystals from a metal chloride.

### Photoelectrochemical properties of InN nanocrystals

**Fig. 5:**
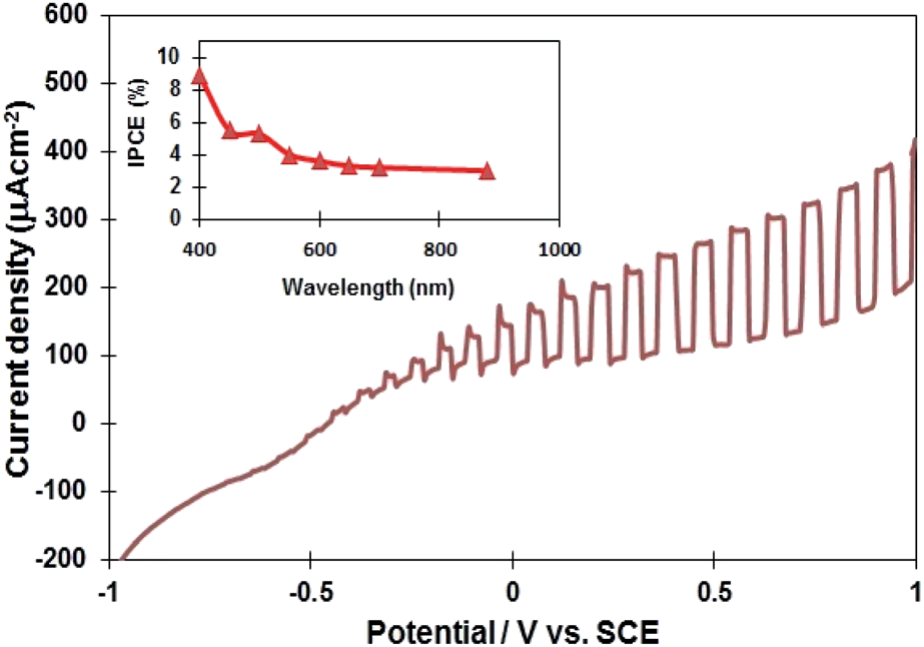
A current potential curve of the InN electrode under Xe lamp illumination (the InN sample synthesized at 450 °C). The inset shows IPCE as a function of wavelength for the InN electrode.

Several InN electrodes synthesized at 400 °C, 450 °C, and 500 °C were prepared to examine their photoelectrochemical properties. The current–potential curve of the InN electrode synthesized at 450 °C is presented in Fig. 5 (the curves for the 400 °C and 500 °C samples are shown in Fig. S7†). The results indicated that all the InN electrodes contained n-type semiconductors. Typically, the onset potential of the InN sample synthesized at 450 °C was found to be −0.72 V *vs.* SCE, and the photocurrent density at 1.0 V was 406 μA cm^−2^, which was found to be the highest among the three samples (Table 2). InN is normally doped with oxygen impurities, which make it n-type. Therefore, the photoresponse observed for all samples was thought to originate from the InN electrodes.

**Table 2:** The photoelectrochemical properties of InN nanocrystals

InN sample	Onset potential *V*_on_ (V *vs.* SCE)	Photocurrent density (μA cm^−2^) (at 1.0 V)	Donor density (*N*_D_) (10^20^ cm)
400 °C	−0.75	361.8	7.86
450 °C	−0.72	406.0	5.62
500 °C	−0.71	380.8	7.89

To understand the carrier transport of InN electrodes, we compared the donor density of the InN samples using Mott–Schottky plots (Fig. S8†). As summarized in Table 2, InN samples synthesized at 450 °C had the lowest donor density, which probably led to the improvement of the photocurrent in the InN electrode.

The inset in Fig. 5 displays the quantum efficiency of the InN electrode synthesized at 450 °C. The photoelectrochemical cell consisting of the InN nanocrystals exhibited an IPCE response in the visible and near-IR region (400–880 nm). The IPCE was estimated to be 9% at 400 nm and 3.6% at 880 nm. Lindgren *et al.*^27^ reported that the maximum IPCE of InN thin film electrodes was 2% at 350 nm, whereas no photoresponse was observed in the visible region. It is worth noting that responses in the visible and near-IR regions were observed for the first time using our nanostructured InN electrode material. In addition, we have compared the photocurrent of InN electrodes using a reported data.^27^ For our experiments (Table 2), the observed photocurrents for the InN electrodes synthesized at 400 °C, 450 °C, and 500 °C were 297.1 μA cm^−2^, 323.9 μA cm^−2^, and 297.1 μA cm^−2^ at 0.7 V, respectively. Lindgren *et al.*^27^ reported the DC magnetron reactive sputtered method to obtain an InN thin-film electrode at 425 °C. Agglomerated InN nanocrystals consisted of two phases of InN and In_2_O_3_ formed during annealing process. The photocurrent of InN electrode was 33 μA cm^−2^ at 0.7 V under Xe lamp irradiation, which was much lower than those of our InN electrodes. Our nitridation-based molten salt may provide a single-phase InN, leading to the higher photocurrent density.

## Conclusions

For the first time, we synthesized single-phase InN nanocrystals through the reaction of InCl_3_ and LiNH_2_ using LiCl–KCl as the molten salt. The InN nanocrystals were successfully obtained at relatively low temperatures ranging from 400 °C to 500 °C. The use of a molten salt could accelerate the homogeneous formation of InN nanocrystals with sizes of 50–60 nm. Notably, the photoelectrochemical cell fabricated using the InN nanocrystals exhibited an IPCE response in the visible and near-IR region (400–880 nm). The precise control of the growth of InN nanocrystals using molten salt-assisted nitridation will shed light on a new generalized approach to create versatile nitride nanocrystals.

## Conflicts of interest

There are no conflicts to declare.

## Supplementary Material

RA-010-D0RA07172B-s001
