# Supplementary material for: A molten salt-based nitridation approach for synthesizing nanostructured InN electrode materials
Source: RSC Adv. 2020 Oct 12;10(61):37576–81. doi: 10.1039/d0ra07172b (PMC9057135; doi:10.1039/d0ra07172b)
Supplement: RA-010-D0RA07172B-s001 [file RA-010-D0RA07172B-s001.pdf]

Electronic Supplementary Material (ESI) for RSC Advances.
This journal is © The Royal Society of Chemistry 2020

Supporting Information

A molten salt-based nitridation approach for synthesizing nanostructured InN electrode materials

Gani Purwiandono,[1,2] Kazuhiro Manseki[1*] and Takashi Sugiura[1]

[1]*The Graduate School of Natural Science and Technology, Gifu University, 501-1193, Japan*

[2]*Department of Chemistry, Universitas Islam Indonesia, 55584, Indonesia*

Contents

Table S1: The melting points of LiCl-KCl molten salt systems.

Fig. S1: XPS spectra of InN samples: (a) whole region, (b) In 3d, (c) Li 1s, (d) Cl 2p and (e) K 2p.

Fig. S2: SEM images of InN crystals prepared by the reaction of $InCl_3$, $LiNH_2$ and LiCl-KCl at (a) 350°C, (b) 400°C, (c) 450°C, (d) 500°C and (e) 550°C

Fig. S3: Brunauer-Emmett-Teller (BET) isotherms and Barret-Joyner-Halenda (BJH) pore-size distribution (inset) of InN synthesized at 400°C, 450°C and 500°C.

Fig. S4: XRD patterns of InN synthesized using $InCl_3$ and $LiNH_2$ without LiCl-KCl. The reactions were carried out at 400°C, 450°C, 500°C and 550°C.

Fig. S5: SEM images of InN synthesized using $InCl_3$ and $LiNH_2$ without LiCl-KCl at (a) 400°C, (b) 450°C, (c) 500°C and (d) 550°C.

Fig. S6: (a) Absorption spectra and (b) band gap determination of InN thin films.

Fig. S7: Current potential curves of the InN electrodes under Xe lamp illumination with 64R cut filter.

Fig. S8: Mott-Schottky plots of InN electrodes for 400°C, 450°C and 500°C samples. Donor densities (N_D) are also presented in the figure.

Table S1. The melting points of LiCl-KCl molten salt systems.

Composition of molten salts (molar ratio)		Melting point
LiCl	KCl	
0	100%	770°C
20%	80%	707°C
40%	60%	589°C
60%	40%	353°C
80%	20%	450°C
100%	0	605°C

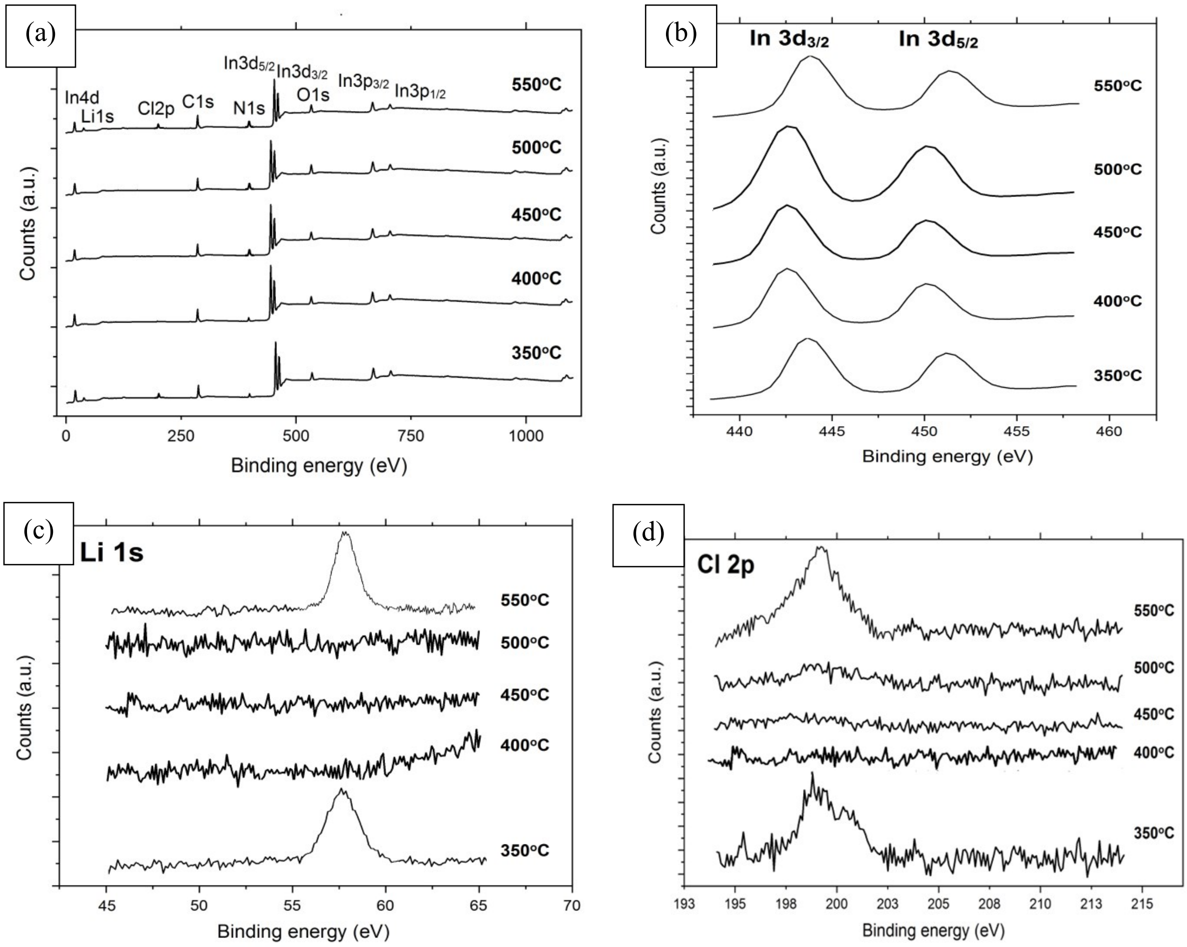

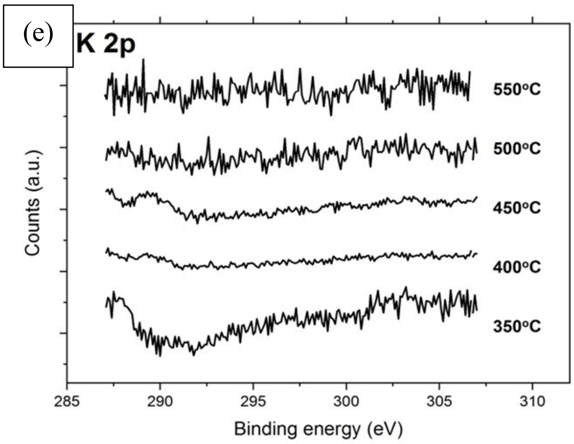

Fig. S1 XPS spectra of InN samples: (a) whole region, (b) In 3d, (c) Li 1s, (d) Cl 2p and (e) K 2p.

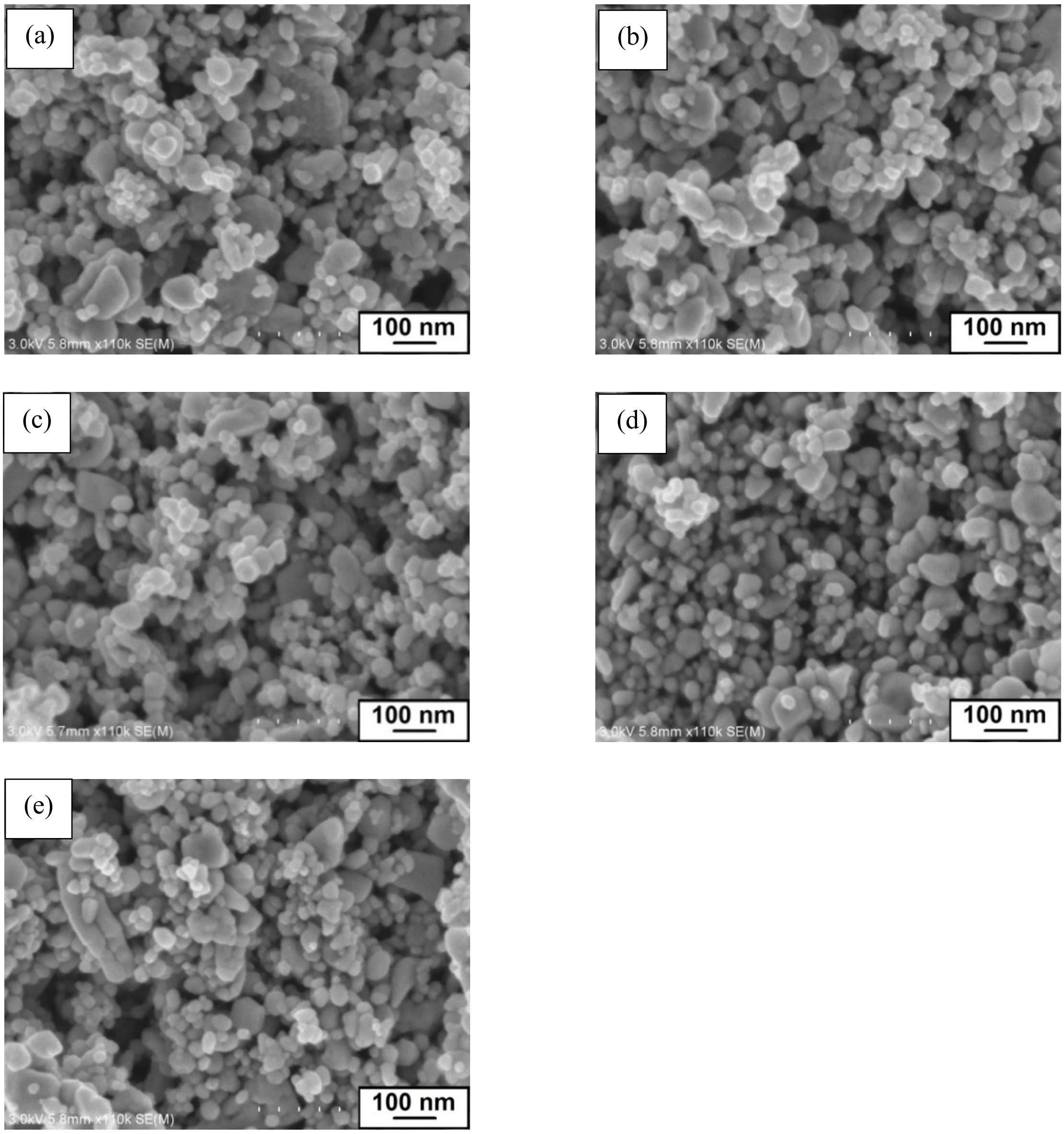

Fig. S2 SEM images of InN crystals prepared by the reaction of $InCl_3$, $LiNH_2$ and LiCl-KCl at (a) 350°C, (b) 400°C, (c) 450°C, (d) 500°C and (e) 550°C.

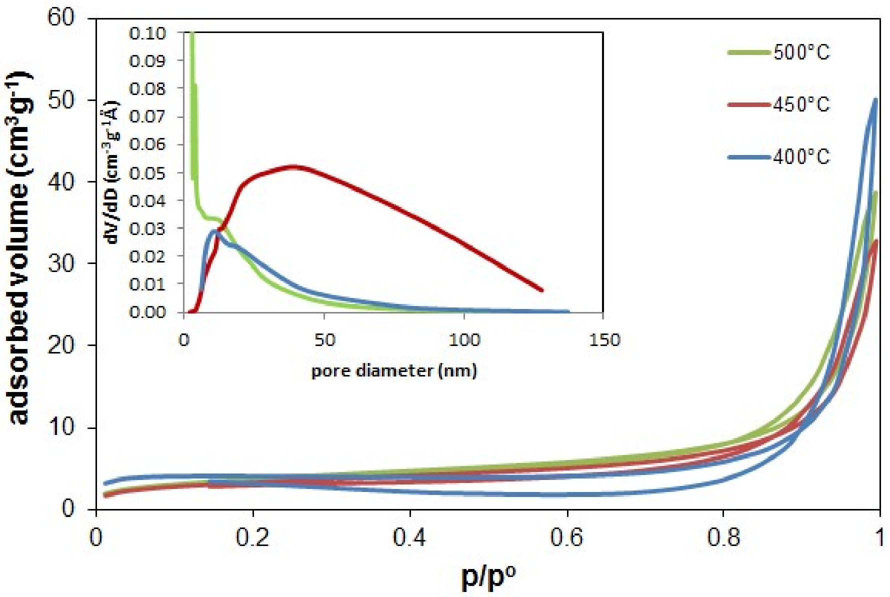

Fig. S3 Brunauer-Emmett-Teller (BET) isotherms and Barret-Joyner-Halenda (BJH) pore-size distribution (inset) of InN synthesized at 400°C, 450°C and 500°C.

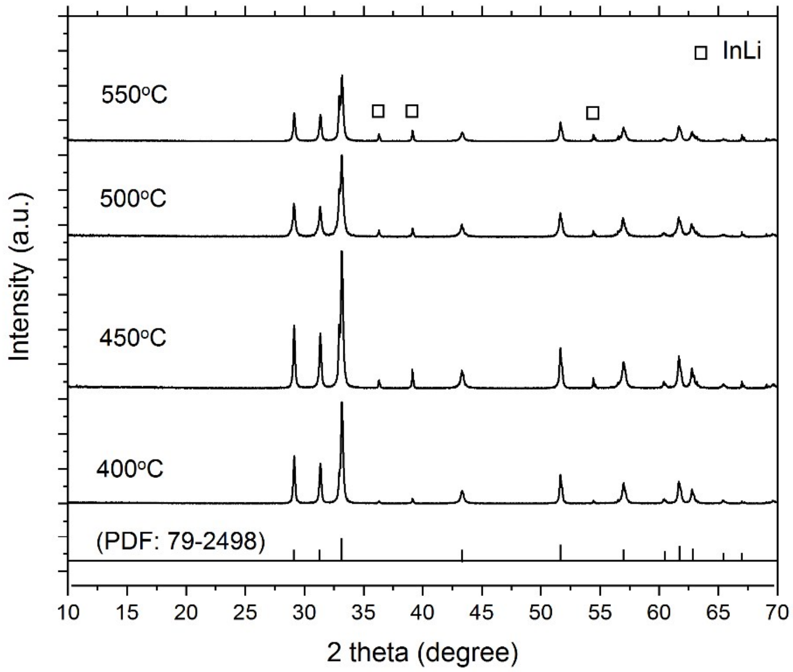

Fig. S4 XRD patterns of InN synthesized using $InCl_3$ and $LiNH_2$ without LiCl-KCl. The reactions were carried out at 400°C, 450°C, 500°C and 550°C.

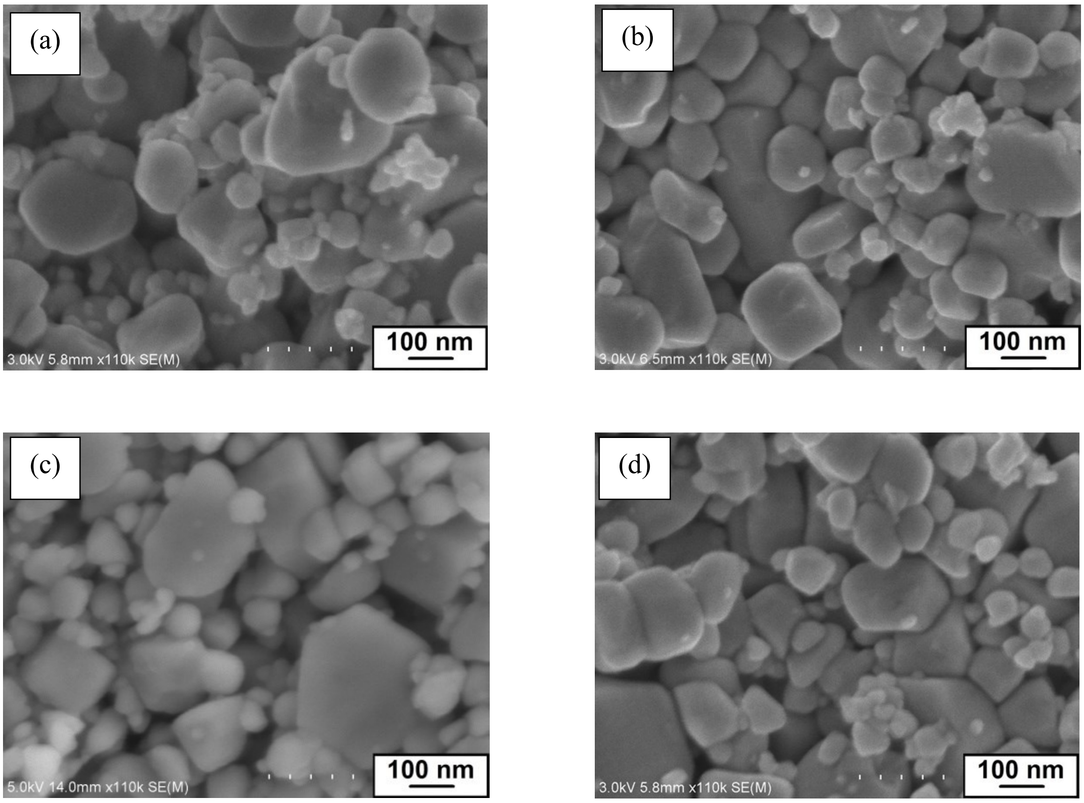

Fig. S5 SEM images of InN synthesized using $InCl_3$ and $LiNH_2$ without LiCl-KCl at (a) 400°C, (b) 450°C, (c) 500°C and (d) 550°C.

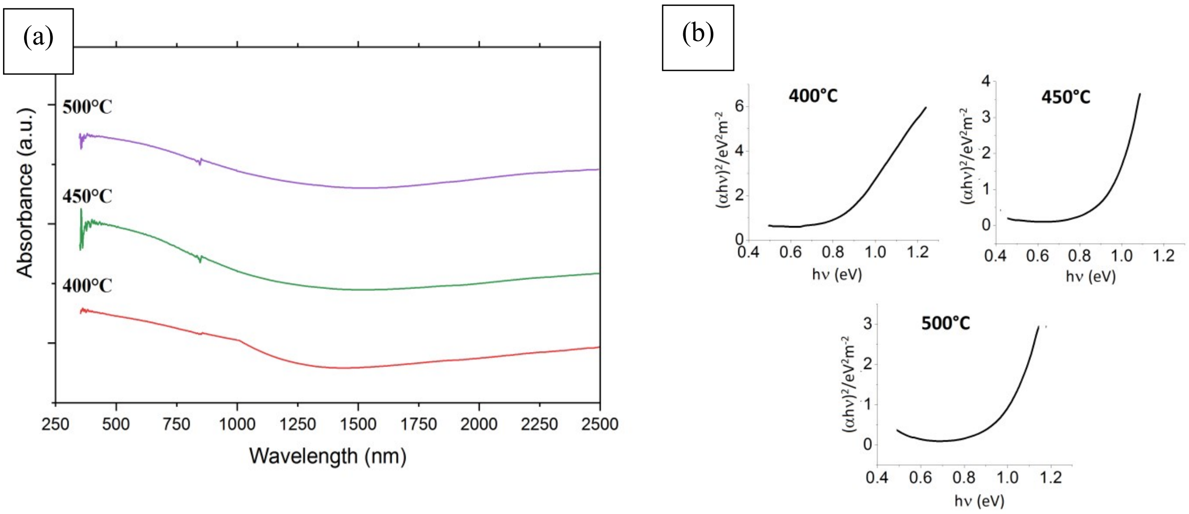

Fig. S6 (a) Absorption spectra and (b) band gap determination of InN thin films.

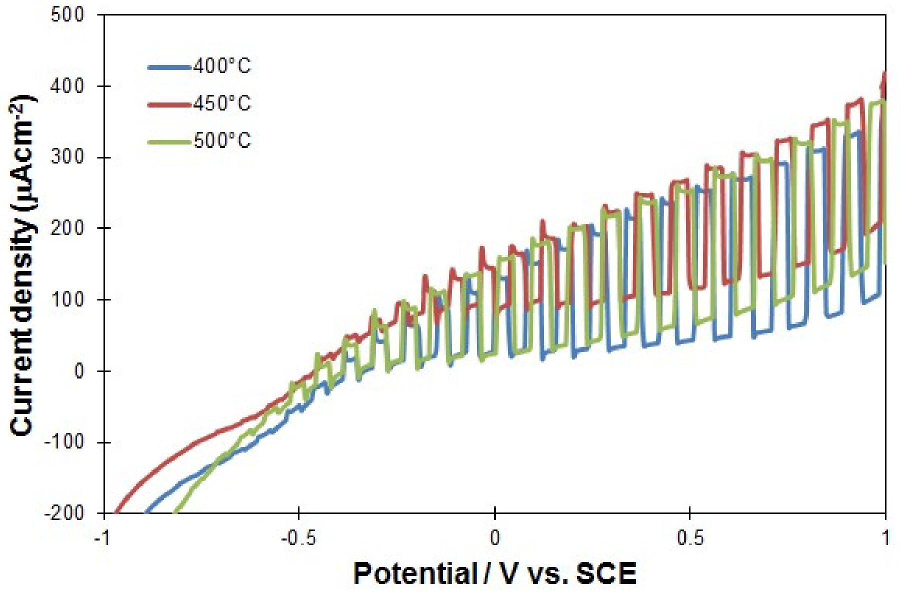

Fig. S7 Current potential curves of the InN electrodes under Xe lamp illumination with 64R cut filter.

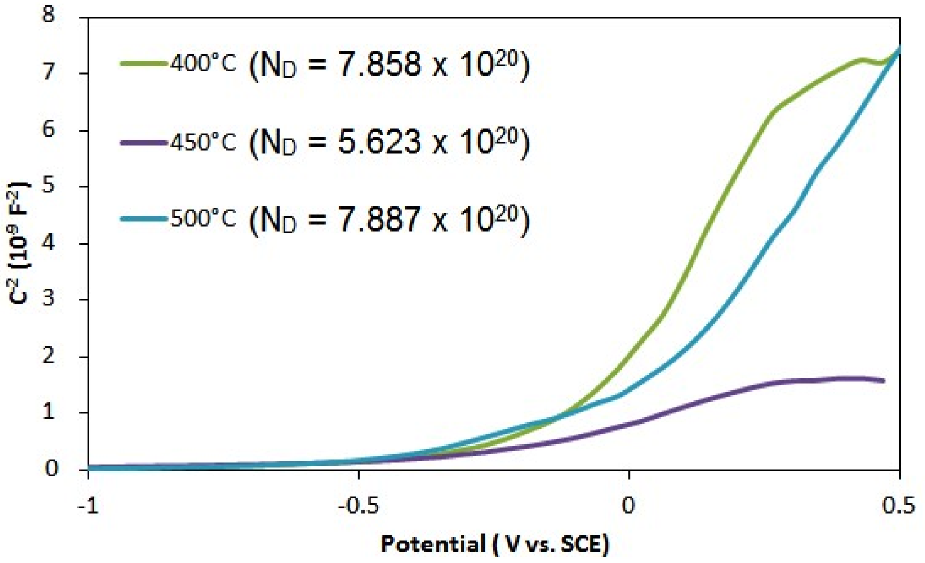

Fig. S8 Mott-Schottky plots of InN electrodes for 400°C, 450°C and 500°C samples. Donor densities (N_D) are also presented in the figure.
